# Loss of Sirt6 in adipocytes impairs the ability of adipose tissue to adapt to intermittent fasting

**DOI:** 10.1038/s12276-021-00664-1

**Published:** 2021-09-07

**Authors:** Dandan Wu, In Hyuk Bang, Byung-Hyun Park, Eun Ju Bae

**Affiliations:** 1 Department of Biochemistry and Molecular Biology, Chonbuk National University Medical School, Jeonju, Jeonbuk 54896 Republic of Korea; 2 College of Pharmacy, Chonbuk National University, Jeonju, Jeonbuk 54896 Republic of Korea

**Keywords:** Obesity, Fat metabolism

## Abstract

Intermittent fasting (IF) is gaining popularity for its effectiveness in improving overall health, including its effectiveness in achieving weight loss and euglycemia. The molecular mechanisms of IF, however, are not well understood. This study investigated the relationship between adipocyte sirtuin 6 (Sirt6) and the metabolic benefits of IF. Adipocyte-specific Sirt6-knockout (aS6KO) mice and wild-type littermates were fed a high-fat diet (HFD) ad libitum for four weeks and then subjected to 12 weeks on a 2:1 IF regimen consisting of two days of feeding followed by one day of fasting. Compared with wild-type mice, aS6KO mice subjected to HFD + IF exhibited a diminished response, as reflected by their glucose and insulin intolerance, reduced energy expenditure and adipose tissue browning, and increased inflammation of white adipose tissue. Sirt6 deficiency in hepatocytes or in myeloid cells did not impair adaptation to IF. Finally, the results indicated that the impaired adipose tissue browning and reduced expression of UCP1 in aS6KO mice were accompanied by downregulation of p38 MAPK/ATF2 signaling. Our findings indicate that Sirt6 in adipocytes is critical to obtaining the improved glucose metabolism and metabolic profiles conferred by IF and that maintaining high levels of Sirt6 in adipocytes may mimic the health benefits of IF.

## Fat metabolism: facilitating responses to intermittent fasting

The health benefits of intermittent fasting regimens are mediated in part by a protein ‘switch’ that modulates metabolic and inflammatory activity in fatty tissue. Several studies have linked such dietary regimens, which entail alternating periods of fasting and eating, to improved metabolic health. Researchers led by Byung-Hyun Park and Eun Ju Bae at Chonbuk National University, Jeonju, South Korea, have now demonstrated that the sirtuin 6 protein coordinates important physiological changes in response to such diets. The researchers noted that intermittent fasting mitigated the effects of a high-fat diet in control mice. In contrast, mice lacking sirtuin 6 in their fat cells exhibited features of metabolic syndrome under these conditions, including active inflammation in fatty tissue and reduced response to insulin. These results suggest that therapies that modulate sirtuin 6 could mirror the benefits achieved through intermittent fasting.

## Introduction

Obesity, which increases the risk of developing metabolic diseases such as type 2 diabetes, is now a global epidemic. Physical activity and dietary manipulation are the effective means of achieving weight loss and treating obesity-related diseases. Calorie restriction (CR) has long been known to cause weight loss and extend the health span of animals^1,2^. Intermittent fasting (IF), also known as intermittent energy restriction, is an eating pattern that cycles between periods of fasting and eating and is gaining popularity as an alternative to CR for its effectiveness in improving overall health^3,4^. Interestingly, a number of studies in animals and humans have demonstrated that the health advantages of IF are, in fact, even more extensive than initially thought^5–7^. The most common IF regimens in humans include alternate-day fasting, twice-weekly fasting, and time-restricted feeding^3^. IF has been shown to promote health and reduce the risk of many chronic diseases, including metabolic syndrome, as well as various cancers, cardiovascular maladies, and neurodegenerative diseases^4,8,9^.

Comprehensive reviews have determined the precise mechanism that links IF and these health advantages. The benefits of IF are derived primarily from a negative energy balance with or without a reduction in calorie intake and consequent weight loss^5,6^. During periods of prolonged fasting or when glycogen stores are depleted in the liver, metabolic switching occurs from liver-originated glucose to adipocyte-derived fatty acids and ketones produced in the liver. Thus, a shift from lipid storage to fat mobilization occurs during IF, and adipose tissue (AT) is the main driver organ responsible for generating fuel sources during fasting. With repeated cycles of fasting and refeeding, IF promotes AT remodeling, which triggers lipolysis, alters the accumulation of immune cells in white AT (WAT), browns white adipocytes, activates brown AT (BAT), and precipitates thermogenesis^10–14^. However, the molecular links that mediate these AT adaptations remain poorly understood.

Sirtuins, as nutrient-sensing NAD^+^-dependent histone deacetylase proteins, are upregulated by fasting or CR and counteract diseases related to caloric excess such as obesity and type 2 diabetes^15,16^. Numerous studies on lower animals and nonhuman primates have shown that sirtuins play a mediating role between CR/IF and subsequently observed lifespan extensions and general health improvements^17^. For example, Sirt1 transgenic (Tg) mice display phenotypes resembling those associated with CR^18^. Natural and synthetic activators of Sirt1 also act as CR mimetics^19,20^. Boutant et al., on the other hand, reported that Sirt1 Tg mice do not exhibit metabolic effects that are similar to or stronger than those associated with IF^21^. This implies that the health benefits conferred by IF and CR are mediated by different mechanisms.

Sirt6 is an alternative member of the mammalian sirtuin family that is critical to controlling metabolism. Sirt6 Tg mice, similar to Sirt1 Tg mice, also exhibit superior protection against diet-induced metabolic stresses^22^, and Sirt6 Tg male mice are long-lived^23^. Although Sirt6 has been reported to mediate the effects of CR in cellular models^24^, the function of Sirt6 in mediating the beneficial effects of IF is unknown. We have previously reported, and other researchers have also found, a tissue-specific role for Sirt6. Liver Sirt6-knockout (KO) mice fed either normal chow or a high-fat diet (HFD) demonstrate exacerbated hepatic steatosis/hepatitis, inflammation, insulin resistance^25,26^, and impaired ketogenesis^27^. Myeloid Sirt6 is critical for preventing HFD-induced obesity and associated AT inflammation^28^. Mice lacking Sirt6 in adipocytes (aS6KO mice) exhibit an obese phenotype and diabetes and are particularly ill-suited for IF with regard to metabolic effects, for example, they experience impairment of lipolysis and thermogenesis, insulin resistance, and adipose tissue inflammation^29–32^.

In this study, we reasoned that adipose Sirt6 may be critical for realizing the metabolic flexibility conferred by IF. To test this hypothesis, we subjected aS6KO mice to IF and compared them with hepatocyte- and myeloid-specific Sirt6 KO IF-subjected mice.

## Materials and methods

### Animals and diet regimens

Eight-week-old male C57BL/6 mice were subdivided into three groups: the first and second groups were fed a normal chow diet (NCD) or HFD ad libitum over a 16-week period of study, while the third group was fed a HFD for the first four weeks and then switched to an IF regimen consisting of two-day feeding/one-day fasting periods (2:1 IF) for the remaining 12 weeks (Supplementary Fig. 1a). aS6KO mice (*Sirt6*^flox/flox^;*Adipoq-Cre*) were generated by crossing *Sirt6*^*flox/flox*^ mice (B6;129-*Sirt6*^tm1Ygu^/J) and *Adipoq-Cre* mice (B6.FVB-Tg(*Adipoq-cre*)1Evdr/J*)* as previously described^30^. Myeloid-^28^ and hepatocyte-specific Sirt6 KO mice^25^ were generated by crossing *Sirt6*^*flox/flox*^ mice (B6;129-*Sirt6*^tm1Ygu^/J) with *LysM-Cre* mice (B6.129P2-*lyz2*^tm(cre)lfo^/) and *Albumin-Cre mice* (B6.Cg-Tg(*alb-Cre*)21Mgn/J), respectively. These three kinds of male Sirt6 KO mice and wild-type littermates were fed a HFD for four weeks and subsequently placed on an IF regimen for 12 weeks. Food intake and body weight were monitored every six days throughout the experiments. All mice were fasted overnight before sacrifice at the end of the study. All experimental mice were housed in a controlled barrier facility (12-h light/dark cycle, 23 ± 1 °C, 60–70% humidity). The study protocol was approved by the Institutional Animal Care and Use Committee of Chonbuk National University (Permit No: CBNU 2020-039).

### Indirect calorimetry

Indirect calorimetry was performed on mice after 12 weeks of IF treatment using an 8-chamber Environment Controlled CLAMS (Oxymax System, Columbus Instruments, Columbus, OH, USA) with one mouse/chamber. The mice were placed in metabolic cages for one day to adapt and avoid stress during analysis. After 24 h of acclimatization, the mice were monitored for 24 h under fed conditions in both the ad libitum and IF groups. Food and water were provided ad libitum during the feeding period, and only water was provided during the fasting period. Locomotor activity was determined at the same time energy expenditure was measured using infrared-beam interruption.

### Core-body temperature

Body temperature was measured in conscious mice using a digital thermometer with a rectal probe (Power-Tronics, Kerrville, TX, USA).

### Glucose- and insulin-tolerance tests

An intraperitoneal glucose-tolerance test (GTT) and insulin-tolerance test (ITT) were performed after 12 weeks of IF. For the GTT, after 16 h of fasting, mice received a glucose solution via intraperitoneal injection at a dose of 1 g/kg body weight. The glucose concentration was evaluated in blood samples collected from the tail at 0 min (baseline) and at 15, 30, 60, 90, and 120 min after glucose injection. For the ITT, blood glucose levels were measured after a 6-h fast followed by intraperitoneal injection with 0.75 units/kg body weight of human insulin (Sigma-Aldrich, St Louis, MO, USA).

### Histology

Adipose tissues were immediately placed in fixative (10% formalin) solution. Histological sections (7 μm) were cut from formalin-fixed paraffin-embedded tissue blocks. Tissue sections were stained with hematoxylin–eosin (H&E) under standard conditions. Immunohistochemical staining was performed using a biotin-free immunoenzymatic antigen-detection system (Abcam, Cambridge, UK). For immunofluorescence staining, the sections were incubated with a combination of anti-perilipin (Fitzgerald, MA, USA) and anti-F4/80 (Abcam) at 4 °C overnight. After incubation with the corresponding fluorochrome-conjugated secondary antibodies, the sections were mounted and visualized using an LSM510 confocal laser-scanning microscope (Carl Zeiss, Oberkochen, Germany). The adipocyte area in the selected fat tissue sections was measured using iSolution DT 36 software (Carl Zeiss).

### Biochemical analysis

Plasma levels of TNF-α and IL-10 were measured by using specific ELISA kits (Invitrogen, Carlsbad, CA, USA). Plasma levels of nonesterified fatty acids (NEFAs) and glycerol were measured using commercially available kits (Wako, Osaka, Japan and Sigma-Aldrich, respectively).

### Flow cytometric analysis

Stromal vascular cells (SVCs) from epididymal WAT were isolated and incubated in FACS buffer containing 2% FBS with Fc Block (BD Biosciences, San Jose, CA, USA) for 30 min at 4 °C prior to staining with antibodies against F4/80 (1 μg/ml), CD11b (0.4 μg/ml), CD11c (0.4 μg/ml), or CD206 (0.4 μg/ml) for 30 min at 4 °C. The primary antibodies were obtained from BD Biosciences. The stained cells were gently washed three times and resuspended in FACS buffer. The SVCs were analyzed using a FACSCalibur instrument (BD Biosciences). Unstained, single-stained, and a fluorescence-minus-one control were used to set the compensation and gates.

### Western blotting

The proteins in tissue homogenates (20 μg) were separated by 10% SDS-PAGE and transferred to PVDF membranes. After blocking with 5% skim milk, the blots were probed with primary antibodies against Sirt6, Akt, p-Akt (Ser473), p38 MAPK, p-p38 MAPK, p-ATGL (Ser406), HSL, p-HSL (Ser563, Ser565, Ser660), CREB, p-CREB (Cell Signaling, Beverly, MA, USA), UCP1, ATGL (Abcam), HSP90 (Enzo Life Sciences, Plymouth Meeting, PA, USA), ATF2, and p-ATF2 (Santa Cruz Biochemicals, Dallas, TX, USA). Immunoreactive bands were detected with a Las-4000 imager (GE Healthcare Life Science, Pittsburgh, PA, USA).

### RNA isolation and real-time quantitative RT-PCR (qPCR)

Total RNA was extracted from frozen adipose tissue using an RNA Iso kit (TaKaRa, Tokyo, Japan). First-strand cDNA was generated using the random hexamer primer provided in the first-strand cDNA synthesis kit (Applied Biosystems, Foster City, CA, USA). Specific primers for each gene were designed using PrimerBank (available at https://pga.mgh.harvard.edu/primerbank) (Supplementary Table 1). qPCR was performed in a final volume of 10 μl containing 10 ng of reverse-transcribed total RNA, 200 nM forward and reverse primers, and PCR master mix. qPCR was performed in a 384-well plate using an ABI Prism 7900HT Sequence Detection System (Applied Biosystems).

### Statistical analysis

The data are expressed as the mean ± standard error of the mean (SEM). Statistical comparisons were performed using one-way analysis of variance followed by Fisher’s *post hoc* analysis. The significance of differences between two groups was determined using Student’s unpaired *t*-test. A *p*-value less than 0.05 was considered to indicate significance.

## Results

### IF increases the expression of Sirt6 in the AT of HFD-fed mice

**Fig. 1:**
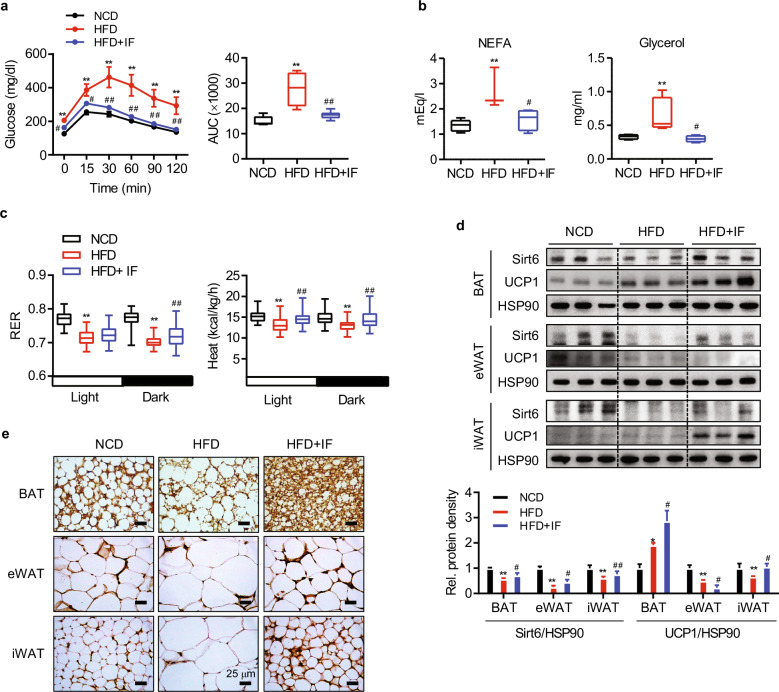
Metabolic characteristics of intermittent HFD-fed mice. Eight-week-old C57BL/6 mice were fed either an NCD or a 60% HFD ad libitum for 16 weeks or subjected to 12 weeks of the 2:1 intermittent fasting (IF) regimen after four weeks of HFD feeding ad libitum. **a** Glucose-tolerance test in mice (*n* = 6). **b** Serum levels of NEFAs and glycerol in mice (*n* = 4). **c** RER (VCO_2_/VO_2_) and heat generation under fed conditions (*n* = 6). **d** Western blot analysis for Sirt6 and UCP1 in adipose tissues. The protein density was quantified (*n* = 3). **e** Immunohistochemical staining of UCP1 in adipose tissues. Scale bar = 25 µm. The values are expressed as the mean ± SEM. ^**^p < 0.01 vs. NCD; ^#^*p* < 0.05 and ^##^*p* < 0.01 vs. HFD. NCD normal chow diet, HFD high-fat diet, AUC area under the curve, NEFA nonesterified fatty acid, BAT brown adipose tissue, eWAT epididymal white adipose tissue, iWAT inguinal white adipose tissue.

In this study, we applied an isocaloric IF regimen to exclude the possibility of weight loss due to reduced caloric intake^33^. Eight-week-old C57BL/6 mice were subdivided into three groups: the NCD, HFD, and HFD + IF groups (Supplementary Fig. 1a). While HFD mice gained body weight more rapidly than NCD mice, HFD + IF mice showed significantly lower body weights than HFD mice during the entire period of IF without changes in food intake (Supplementary Figs. 1b and 1c)^33^. Glucose tolerance in HFD-fed mice was dramatically improved by IF (Fig. 1a). Consistent with the prior finding that basal lipolysis increases with obesity^34^, we observed increased serum levels of NEFAs and glycerol with HFD feeding but amelioration of these changes by IF (Fig. 1b). The lean phenotype in the HFD + IF group was confirmed by H&E staining of liver tissue, BAT, subcutaneous inguinal WAT (iWAT), and epididymal WAT (eWAT), with less fat accumulation in the liver and smaller adipocytes in HFD + IF mice than in HFD mice (Supplementary Fig. 1d). In addition, fewer F4/80^+^ macrophages accumulated in HFD + IF mice than in their HFD counterparts (Supplementary Fig. 1e).

We next performed indirect calorimetry analysis^35^ to measure energy expenditure and to understand the weight loss in the HFD + IF group. Oxygen consumption (VO_2_), CO_2_ production (VCO_2_), the respiratory-exchange ratio (RER, VCO_2_/VCO_2_), and heat generation were lower in HFD mice than in NCD mice, but were significantly enhanced in HFD + IF mice (Fig. 1c and Supplementary Fig. 2), suggesting that the marked weight loss triggered by IF was likely at least partly the result of the increased energy expenditure and thermogenesis. The key characteristic of IF and CR associated with the elevation in whole-body energy expenditure is browning of white adipocytes, namely, development of beige fat^10,11,36^. Brown fat cells catabolize lipids to produce heat, a function that is mediated by uncoupling protein 1 (UCP1). Therefore, we next examined the expression of UCP1 by Western blotting and immunohistochemistry in BAT and iWAT and determined that UCP1 was dramatically increased by IF in both BAT and iWAT (Figs. 1d and 1e). In parallel, adipocyte size was reduced by IF in BAT and iWAT as well as in eWAT. Intriguingly, Sirt6 protein levels were upregulated by IF in these three kinds of AT, whereas they were diminished under HFD feeding compared with NCD feeding, demonstrating a selective correlation with UCP1 only in iWAT (Fig. 1d). These results are consistent with previous findings that Sirt6 expression is reduced in the AT of obese subjects^29,30^ and increased in the AT of individuals with weight loss^37^. Protein expression of other members of the sirtuin family in AT remained unchanged or reduced in response to IF (Supplementary Fig. 3). These findings prompted us to investigate the potential role of Sirt6 in mediating adipose tissue and systemic metabolism under IF conditions in adipocytes.

### The metabolic benefits of IF are attenuated by adipose Sirt6 ablation

**Fig. 2:**
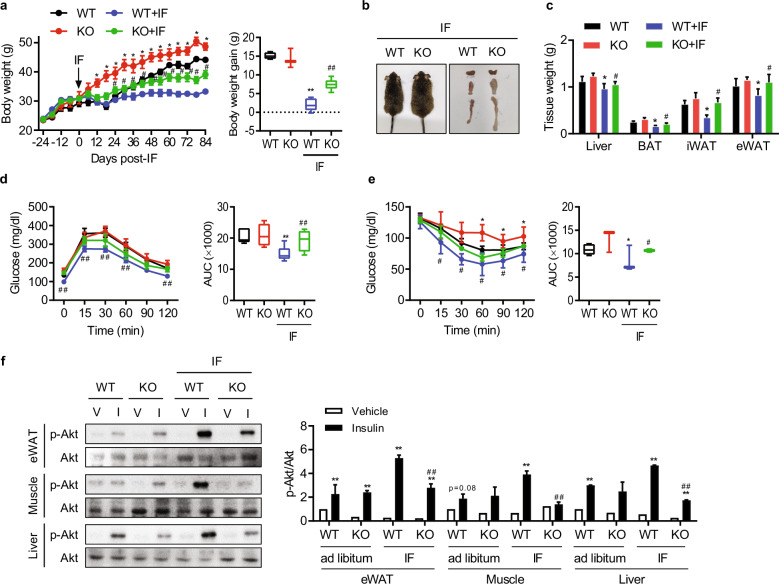
Worsening of insulin resistance in intermittent HFD-fed adipocyte-specific Sirt6-knockout (aS6KO) mice. **a** Body weight changes during the whole 16 weeks of the study (left panel) and body weight gain for 12 weeks after the IF regimen (right panel) in aS6KO (KO) and wild-type (WT) mice (*n* = 5). **b** Gross morphology of mice and adipose tissues. **c** Tissue weights. **d, e** Glucose- and insulin-tolerance tests in mice (*n* = 5). **f** Mice were injected with insulin (0.75 units/kg body weight), and tissues were collected at 5 min (liver) or 10 min (eWAT and skeletal muscle) for Akt phosphorylation (*n* = 3). The values are expressed as the mean ± SEM. ^*^p < 0.05 and ^**^p < 0.01 vs. WT or vehicle (V); ^#^*p* < 0.05 and ^##^*p* < 0.01 vs. WT + IF or WT + insulin (I).

We subsequently gave aS6KO mice access to a HFD ad libitum or placed them on a HFD + IF regimen to investigate the association between adipocyte Sirt6 and the metabolic benefits conferred by IF. aS6KO mice fed a HFD maintained a higher body weight than wild-type (WT) mice throughout the entire period of our study (Fig. 2a, left panel). We evaluated the net changes in body weights during 12 weeks of the IF diet regimen, noting that body weight gain was not different between WT and aS6KO mice fed a HFD ad libitum (15.1 ± 0.44 g vs 14.2 ± 1.5 g) but differed significantly in those subjected to IF (1.97 ± 0.67 vs 7.43 ± 0.60 g) (Fig. 2a, right panel). The gross morphologies and weights of the liver and AT from different fat depots in mice under the IF regimen were consistent with the measured body weights (Figs. 2b and 2c). When GTTs and ITTs were performed, IF was found to have improved glucose intolerance and insulin resistance in WT mice but not in aS6KO mice (Figs. 2d and 2e). Contrary to this observation, mice lacking Sirt6 in hepatocytes or myeloid cells exhibited similar weight increases and glucose and insulin intolerance as WT mice undergoing IF (Supplementary Figs. 4a–4d). This suggests that hepatocyte Sirt6 and myeloid Sirt6, unlike adipocyte Sirt6, are not directly involved in metabolic changes associated with IF.

To measure the direct effects of adipose Sirt6 deficiency on systemic insulin action, we injected WT and aS6KO mice with insulin. Insulin-stimulated Akt phosphorylation in eWAT, skeletal muscle, and liver tissue was similar between the genotypes under HFD ad libitum feeding (Fig. 2f). However, under IF, the insulin-mediated stimulation of p-Akt levels was markedly attenuated in the tissues of aS6KO mice compared to those of WT mice. These results are consistent with the GTT and ITT results, and the data in the aggregate suggest reduced metabolic adaptation upon IF in aS6KO mice.

**Fig. 3:**
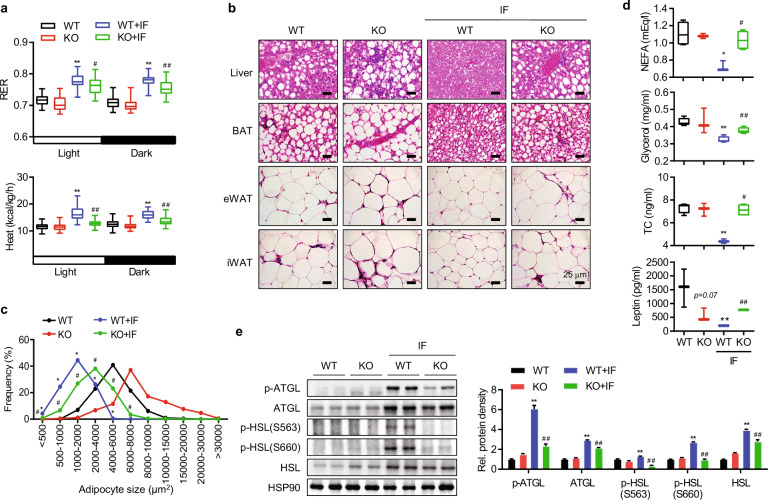
Attenuation of the metabolic benefits of IF in adipocyte-specific Sirt6-knockout (aS6KO) mice. a Indirect calorimetry under fed conditions after 16 weeks of the diet regimen (*n* = 4). **b** H&E-stained sections of liver and adipose tissues. Scale bar = 25 µm. **c** Adipocyte size was measured (*n* = 5). **d** Serum levels of NEFAs, glycerol, total cholesterol (TC), and leptin (*n* = 4). **e** Epididymal fat tissues were subjected to Western blotting. The values are expressed as the mean ± SEM. ^*^p < 0.05 and ^**^p < 0.01 vs. WT; ^#^*p* < 0.05 and ^##^*p* < 0.01 vs. WT + IF. BAT brown adipose tissue, eWAT epididymal white adipose tissue, iWAT inguinal white adipose tissue.

We then repeated indirect calorimetry analysis in aS6KO mice and determined that the RER, oxygen consumption, carbon dioxide production, and heat generation were enhanced with IF in WT mice but significantly decreased in aS6KO mice (Fig. 3a and Supplementary Figs. 5a–5c). Next, H&E-stained sections of liver and ATs were examined. IF significantly improved the severe steatosis from the HFD in WT mice but not in aS6KO mice (Fig. 3b). IF-induced adipocyte-size reduction was minimal in aS6KO mice (Figs. 3b and 3c). IF reduced the serum levels of NEFAs, glycerol, and total cholesterol in WT mice but not in aS6KO mice (Fig. 3d). The serum level of leptin was also downregulated by IF but was significantly higher in aS6KO mice than in WT mice (Fig. 3d). Serum adiponectin levels remained unchanged between genotypes after IF (Supplementary Fig. 5d). The expression of lipolysis-related genes (i.e., ATGL and HSL) and their phosphorylation status was measured by Western blotting, and all markers were found to be suppressed in aS6KO mice (Fig. 3e).

Collectively, these data indicate that adipocyte Sirt6 enhances oxidative capacity and thermogenesis and is necessary for the benefits of IF—protection against HFD-induced obesity and insulin resistance—to accrue to mice.

### Sirt6 deficiency in adipocytes impairs IF-mediated resolution of adipose tissue inflammation

**Fig. 4:**
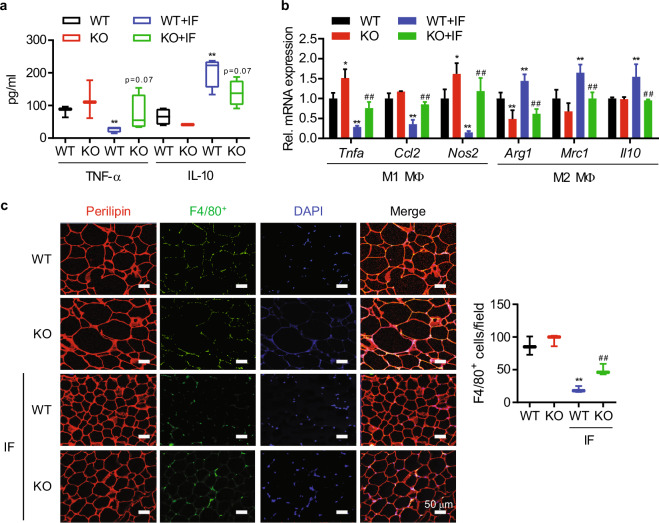
Increases in inflammation and macrophage accumulation in adipocyte-specific Sirt6-knockout (aS6KO) mice. **a** The serum levels of TNF-α and IL-10 were analyzed in WT or aS6KO mice under the HFD + IF regimen (*n* = 4). **b** mRNA expression of genes related to M1 and M2 macrophages as determined by qPCR (*n* = 6). **c** eWAT was immunostained with antibodies against perilipin and F4/80. Scale bar = 50 µm. F4/80-positive cells were quantified (*n* = 7). The values are expressed as the mean ± SEM. ^*^p < 0.05 and ^**^p < 0.01; vs. WT; ^#^*p* < 0.05 and ^##^*p* < 0.01 vs. WT + IF.

Obesity is commonly associated with inflammation in WAT, which causes systemic insulin resistance through the release of various inflammatory cytokines. CR/IF, in contrast, reduces tissue inflammation by altering the subpopulations of macrophages^38^. Therefore, we next determined the serum levels of representative pro- and anti-inflammatory cytokines and macrophage accumulation in the WATs of mice. The serum levels of TNFα and IL-10 were higher and lower, respectively, in aS6KO mice than in WT mice upon IF and were similar between genotypes under HFD feeding (Fig. 4a). qPCR analyses showed that in aS6KO mice, the mRNA levels of various genes specific to M1-like macrophages (*Tnfa, Il1b, Ccl2*, and *Nos2*) were upregulated, while those specific to M2-like cells (*Arg1*, *Mrc1*, and *Il10*) were downregulated (Fig. 4b). Immunofluorescence staining of eWAT indicated the increased infiltration of F4/80-positive macrophages in aS6KO mice (Fig. 4c). Flow cytometry analysis also revealed a higher percentage of M1-like macrophages (F4/80^+^CD11b^+^ CD11c^+^) in the eWAT of aS6KO mice than in that of WT mice (Supplementary Fig. 6). Taken together, these results suggest that Sirt6 deficiency in adipocytes leads to an increased inflammatory response in WAT under the IF regimen.

### Sirt6 deficiency impairs IF-mediated browning of white adipocytes

**Fig. 5:**
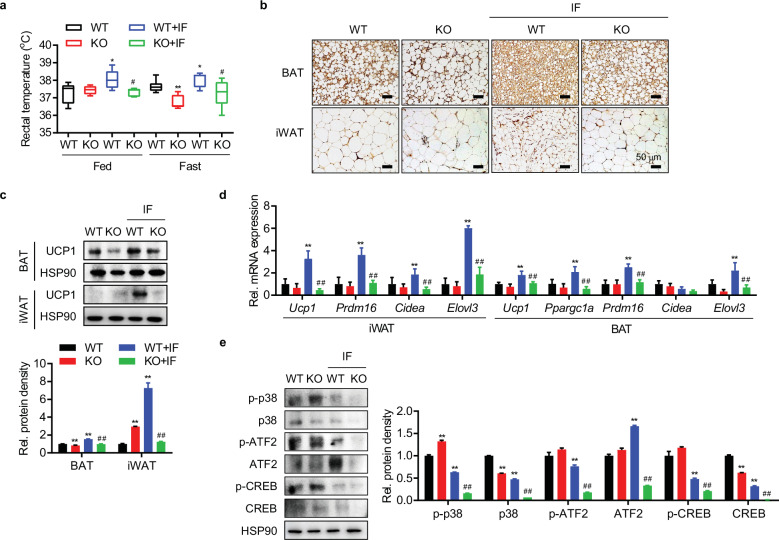
Impairment of IF-induced adipose browning and thermogenesis in adipocyte Sirt6-knockout (aS6KO) mice. **a** Rectal temperatures of mice under the HFD + IF regimen measured under fed or fasting conditions (*n* = 6). **b** Immunohistochemical staining of UCP1 in BAT and iWAT. Scale bar = 50 µm. **c** Western blot analysis of UCP1 expression in adipose tissue (*n* = 3). **d** mRNA expression of adipose tissue browning genes (*n* = 6). **e** Western blot analysis of p38, ATF2, and CREB expression in iWAT. The values are expressed as the mean ± SEM. *p < 0.05 and **p < 0.01 vs. WT; ^#^*p* < 0.05 and ^##^*p* < 0.01 vs. WT + IF.

In a study involving C57BL/6 mice, we demonstrated that IF increased energy expenditure and adipose browning (Figs. 1c–1e). Therefore, we compared the IF-promoted browning ability of WT and aS6KO mice. Consistent with their heavier body weight, aS6KO mice displayed a lower rectal temperature than WT mice upon IF, reflecting their lower energy expenditure and heat generation (Fig. 5a). As assessed by immunostaining and Western blotting analyses for UCP1 in BAT and iWAT, IF-induced beiging of iWAT was prominent, while BAT activation was relatively mild (Figs. 5b and 5c). A lower level of UCP1 with a larger adipocyte size was observed in both BAT and iWAT in aS6KO mice compared with WT animals upon IF, but a striking reduction in UCP1 protein by Sirt6 deficiency was observed only in iWAT (Fig. 5c). The mRNA levels of beige/brown adipocyte marker genes (e.g., *Ucp1*, *Ppargc1a*, *Prdm16*, *Cidea*, and *Elovl3*) were significantly downregulated in the BATs and iWATs of aS6KO mice compared to those of WT mice (Fig. 5d).

IF increases sympathetic activity^39,40^, and the corresponding increases in catecholamine levels activate β3-receptor-dependent signaling in BAT and WAT, which promotes a thermogenic response. When the representative downstream signaling molecules related to beiging were examined in iWAT, the levels of phospho-p38 MAPK and the total and phosphorylated forms of ATF2 and CREB were significantly lower in aS6KO mice than in WT mice upon IF (Fig. 5e). Of interest, the levels of these signaling molecules in BAT were not different between genotypes (Supplementary Fig. 7a), indicating an iWAT-specific response. Hepatic expression of gluconeogenic genes did not show significant differences either between genotypes or between diet regimens (Supplementary Fig. 7b).

## Discussion

Diminished fat browning and increased AT inflammation are key characteristics of obesity in animals and humans. Both are associated with glucose intolerance and insulin resistance. Intermittent fasting improves these phenotypes. Until now, the cellular and molecular links mediating IF and the associated phenotypic changes have remained poorly understood. We confirmed in this study that a 2:1 IF regimen protected mice from diet-induced obesity and metabolic disturbances without reducing food intake. These outcomes were accompanied by significant induction of Sirt6 protein expression in BAT, eWAT, and iWAT, implying a potential role of Sirt6 in metabolic adaptations to IF. Specifically, our study suggests that Sirt6 in adipocytes is a crucial player in IF-promoted metabolic adaptations. By mimicking the benefit of the IF diet regimen, activation of Sirt6 could serve as a defense against obesity and type 2 diabetes.

What was responsible for the observed improvement gaps between WT and aS6KO mice subjected to IF? The answer involves alterations in both WAT and BAT function, and several mechanisms are possible. First, enhanced adipose inflammation in eWAT could have led to the diminished IF response observed in aS6KO mice in terms of glucose and insulin responses. Obesity and type 2 diabetes are tightly linked to increased inflammation and M1 macrophage accumulation in WAT^41^, whereas CR and IF have been found to suppress tissue inflammation in humans and animals^33,38,42,43^, although the latter finding is controversial^44,45^. CR/IF or acute fasting has been shown to either increase or decrease WAT macrophage numbers and inflammatory cytokine levels, and these differences could possibly be due to the degree of AT lipolysis, depending on the fasting period. Recently, a 2:1 IF regimen in HFD-fed mice was shown to reduce the number of M1 macrophages in eWAT by increasing AT vascular endothelial growth factor levels^33^. Consistent with this report, our current findings showed (1) decreased numbers of total and M1 macrophages in the eWAT of HFD + IF mice (Supplementary Fig. 1e), (2) high numbers of M1 macrophages and high levels of related genes in the eWAT of aS6KO mice, and (3) lower serum levels of the anti-inflammatory cytokine IL-10 in aS6KO mice than in WT mice. These results are consistent with our previous report showing that adipocyte Sirt6 decreases the M1 composition in eWAT^30^. The question of how adipocyte Sirt6 regulates the AT macrophage subpopulation under IF conditions requires further study.

Second, insulin-stimulated phosphorylation of Akt in eWAT, muscle, and liver tissue was found to be lower in aS6KO mice than in WT mice, suggesting decreased insulin sensitivity in aS6KO mice under IF. A previous study showed that CR preferentially stimulated glucose uptake in white fat without affecting it in BAT or muscle^36^, suggesting that white fat depots are major tissues of glucose disposal affected by CR. Therefore, the impairment of glucose homeostasis in aS6KO mice subjected to IF could be mainly attributable to eWAT with features of inflammation and insulin resistance, although decreased phosphorylation of Akt was also observed in the skeletal muscle and liver tissue of aS6KO mice under IF. Because eWAT inflammation was evident in the aS6KO mice we used in this study, adipose tissue-derived proinflammatory cytokines might act as a “second hit” for the development of insulin resistance in the skeletal muscle and liver tissues of aS6KO mice.

Third, decreases in beige fat development and BAT activation and the consequent repression of thermogenesis could have led to the increased body weight and insulin resistance observed in aS6KO mice. Previous studies have shown that increased beige and brown fat development enhances energy expenditure and boosts insulin sensitivity^46^, and that IF-mediated metabolic improvement is attributable to adipose thermogenesis^33^. We observed in the current study that (1) in normal mice, IF induced UCP1 protein expression to a similar degree in BAT and iWAT (Fig. 1d), and (2) in aS6KO mice, UCP1 protein expression in both BAT and iWAT was lower than that in WT mice, with a complete reduction in UCP1 expression in iWAT, supporting a selective role for Sirt6 in WAT browning (Fig. 5c). We found that Sirt6 protein expression correlated well with UCP1 expression only in iWAT (Fig. 1d) and that the impairment of the adaptive response to IF in aS6KO mice was severe only in iWAT, not in BAT (Fig. 5c), suggesting an adipose depot-specific response. While both BAT activation and WAT browning contribute to thermogenesis and energy expenditure, a few studies have suggested selective browning of WAT^10,47,48^. It is possible that the underlying thermogenesis mechanism differs depending on the context. BAT is highly efficient and active in heat generation due to its constitutively high expression of UCP1 relative to that in WAT in mice.

Yao et al. previously reported that fat Sirt6 expression is markedly induced by cold as well as β-3 adrenergic agonist treatment and is critical for thermogenesis of brown and beige fat^32^. They showed that, mechanistically, Sirt6 interacts with phospho-ATF2 on the PGC-1α gene promoter to activate its expression and, in turn, increases UCP1 transcription. The requisite role of Sirt6 in the thermogenic response is similar between BAT and WAT, supporting the viability of the β-3 adrenergic receptor/PGC1α/UCP1 axis. In this study, we observed that IF increased the phosphorylation of p38 MAPK, an upstream kinase for ATF2^49^, and that this event was almost completely reduced in the iWAT of aS6KO mice. Concomitantly, the total expression of ATF2 was also suppressed in these mice. These results indicate that Sirt6 regulates UCP1 expression via p38 MAPK/ATF2 signaling in iWAT upon IF.

Interestingly, ATF2 phosphorylation in BAT did not differ between WT and aS6KO mice (Supplementary Fig. 7a), despite the similar extent of UCP1 reduction in aS6KO mice in response to IF. Although we did not address the detailed mechanism of Sirt6 regulation of UCP1 induction in BAT, these results imply that there must be a difference in the intrinsic signaling pathway that leads to UCP1 expression in BAT.

Lipolysis in adipose tissue is tightly regulated by a series of complex mechanisms involving ATGL and HSL, as well as hormonal and biochemical signals that regulate the activity of these lipases. Deletion of adipose lipases results in excess storage of triglycerides, causing adipocyte hypertrophy and obesity^50,51^, whereas overexpression of these enzymes promotes lipolysis^52^. The impaired lipolysis pathway and adipocyte hypertrophy in aSKO mice under IF was attributable to reduced expression of phospho-ATGL, ATGL, phospho-HSL, and HSL. While it has been reported that Sirt6 in adipocytes upregulates ATGL transcription through FoxO1 activation^29^, we did not observe reduced expression of ATGL in aS6KO-HFD mice (Fig. 3e). We cannot explain what caused this difference, but the longer periods of HFD feeding in the mice (eight weeks in the study by Kuang et al. vs 16 weeks in our study) might have contributed to the lack of difference in ATGL expression between WT and aS6KO mice. Instead, we noted a marked reduction in the phosphorylation of lipases upon IF, suggesting that the hormonal and biochemical environment was altered by Sirt6 deficiency. Future studies are needed to determine the mechanism by which Sirt6 ablation decreases the phosphorylation levels of ATGL and HSL.

Despite the marked elevations in the phosphorylation of ATGL and HSL in iWAT caused by IF, the serum levels of NEFAs and glycerol were consistently decreased by IF relative to HFD ad libitum feeding (Fig. 1b and Fig. 3d). The reason for this discrepancy is unclear, but there may be a dissociation between obesity and lipolysis. The association between lipolysis and adipose hypertrophy or obesity remains inconsistent across studies, with both defects^53,54^ and increases in lipolysis^55^ being reportedly linked with obesity.

In summary, adipose Sirt6 is necessary for metabolic adaptation to IF, enhances adipose tissue browning, ameliorates adipose tissue inflammation, and thereby improves insulin action on peripheral tissues, as depicted in Supplementary Fig. 8. Genetic or pharmacological activation of Sirt6 might be an alternative means of achieving the health advantages conferred by IF.

## Supplementary information

Supplementary Information
