# Author Correction: Loss of Sirt6 in adipocytes impairs the ability of adipose tissue to adapt to intermittent fasting

**DOI:** 10.1038/s12276-026-01685-4

**Published:** 2026-02-26

**Authors:** Dandan Wu, In Hyuk Bang, Byung-Hyun Park, Eun Ju Bae

**Affiliations:** 1 Department of Biochemistry and Molecular Biology, Chonbuk National University Medical School, Jeonju, Jeonbuk 54896 Republic of Korea; 2 College of Pharmacy, Chonbuk National University, Jeonju, Jeonbuk 54896 Republic of Korea

Correction to: *Experimental & Molecular Medicine* 10.1038/s12276-021-00664-1, published online 07 September 2021

After online publication of this article, the authors noticed an error in Fig. 5.

Incorrect Figure 5
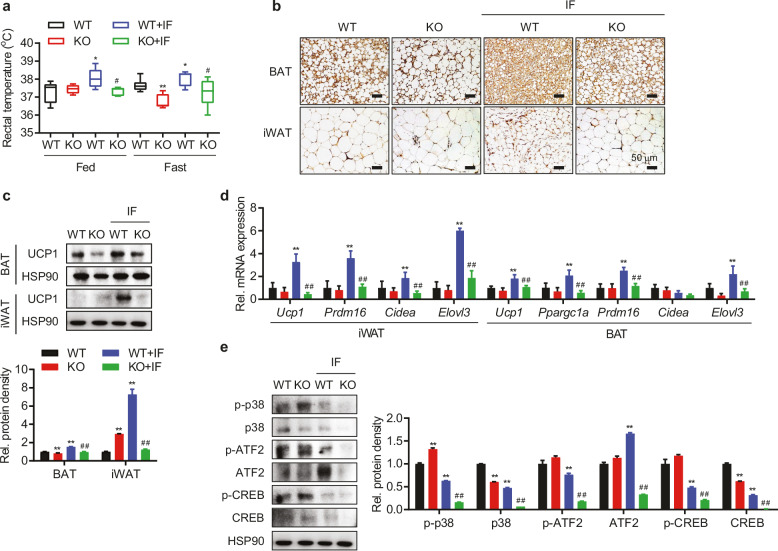


The HSP90 band in panel e has been replaced with a new one, which is shown below.

Corrected Figure 5
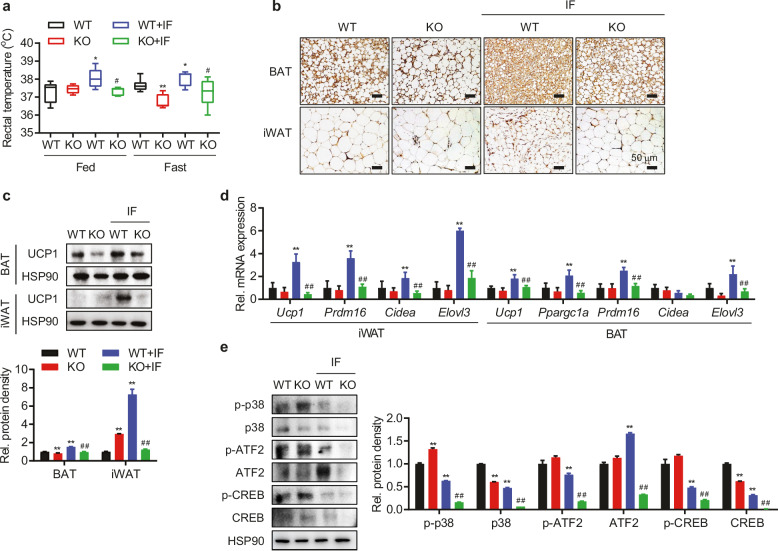


The authors apologize for any inconvenience caused.

The original article has been corrected.

