# Supplementary material for: Loss of Sirt6 in adipocytes impairs the ability of adipose tissue to adapt to intermittent fasting
Source: Exp Mol Med. 2021 Sep 7;53(9):1298–306. doi: 10.1038/s12276-021-00664-1 (PMC8492715; doi:10.1038/s12276-021-00664-1)
Supplement: Supplementary file 1 — Supplementary Information [file 12276_2021_664_MOESM1_ESM.docx]

**Loss of Sirt6 in adipocytes impairs the ability of adipose tissue to adapt to** **intermittent fasting**

Dandan Wu^1^, In Hyuk Bang^1^, Byung-Hyun Park^1,*^, and Eun Ju Bae^2,*^

^1^Department of Biochemistry and Molecular Biology, Chonbuk National University Medical School, Jeonju, Jeonbuk 54896, Republic of Korea

^2^College of Pharmacy, Chonbuk National University, Jeonju, Jeonbuk 54896, Republic of Korea

**^*^Corresponding authors**

Byung-Hyun Park, MD, PhD, 567 Baekje-daero, Deokjin-gu, Jeonju, Jeonbuk 54896, Republic of Korea. Tel.: 82-63-270-3139, E-mail: bhpark@jbnu.ac.kr

Eun Ju Bae, PhD, 567 Baekje-daero, Deokjin-gu, Jeonju, Jeonbuk 54896, Republic of Korea. Tel.: 82-10-3362-4584, E-mail: ejbae7@ jbnu.ac.kr

**Contents**

1. Supplementary table

2. Supplementary figures

**Supplementary Table 1**. Sequences and accession numbers for primers (forward, FOR; reverse, REV) used in qPCR

| Gene | Sequences for primers | Accession No. |
| --- | --- | --- |
| *Nos2* | FOR: TTCTGTGCTGTCCCAGTGAG | NM_010927 |
|  | REV: TGAAGAAAACCCCTTGTGCT |  |
| *Tnfa* | FOR: AGGGTCTGGGCCATAGAACT | NM_ 013693 |
|  | REV: CCACCACGCTCTTCTGTCTAC |  |
| *Ccl2* | FOR: ATTGGGATCATCTTGCTGGT | NM_ 011333 |
|  | REV: CCTGCTGTTCACAGTTGCC |  |
| *Il1b* | FOR: GGTCAAAGGTTTGGAAGCAG | NM_ 008361 |
|  | REV: TGTGAAATGCCACCTTTTGA |  |
| *Il10* | FOR: ATGAACCGAAGCACACCATAG | NM_ 010548 |
|  | REV: ATCAGAGAGTTGACCGCAGTTG |  |
| *Mrc1* | FOR: CTCGTGGATCTCCGTGACAC | NM_ 008625 |
|  | REV: GCAAATGGAGCCGTCTGTGC |  |
| *Arg1* | FOR: TTTTTCCAGCAGACCAGCTT | NM_ 007482 |
|  | REV: AGAGATTATCGGAGCGCCTT |  |
| *Ucp1* | FOR: ACTGCCACACCTCCAGTCATT | NM_ 009463.3 |
|  | REV: CTTTGCCTCACTCAGGATTGG |  |
| *Ppargc1a* | FOR: GGATTGAAGTGGTGTAGCGAC | NM_008904 |
|  | REV: GCTCATTGTTGTACTGGTTGGA |  |
| *Prdm16* | FOR: CAGCACGGTGAAGCCATTC | NM_001291029.1 |
|  | REV: GCGTGCATCCGCTTGTG |  |
| *Cidea* | FOR: ATCACAACTGGCCTGGTTACG | NM_ 007702.2 |
|  | REV: TACTACCCGGTGTCCATTTCT |  |
| *Elovl3* | FOR: GATGGTTCTGGGCACCATCTT | NM_007703 |
|  | REV: CGTTGTTGTGTGGCATCCTT |  |

**2. Supplementary figures**


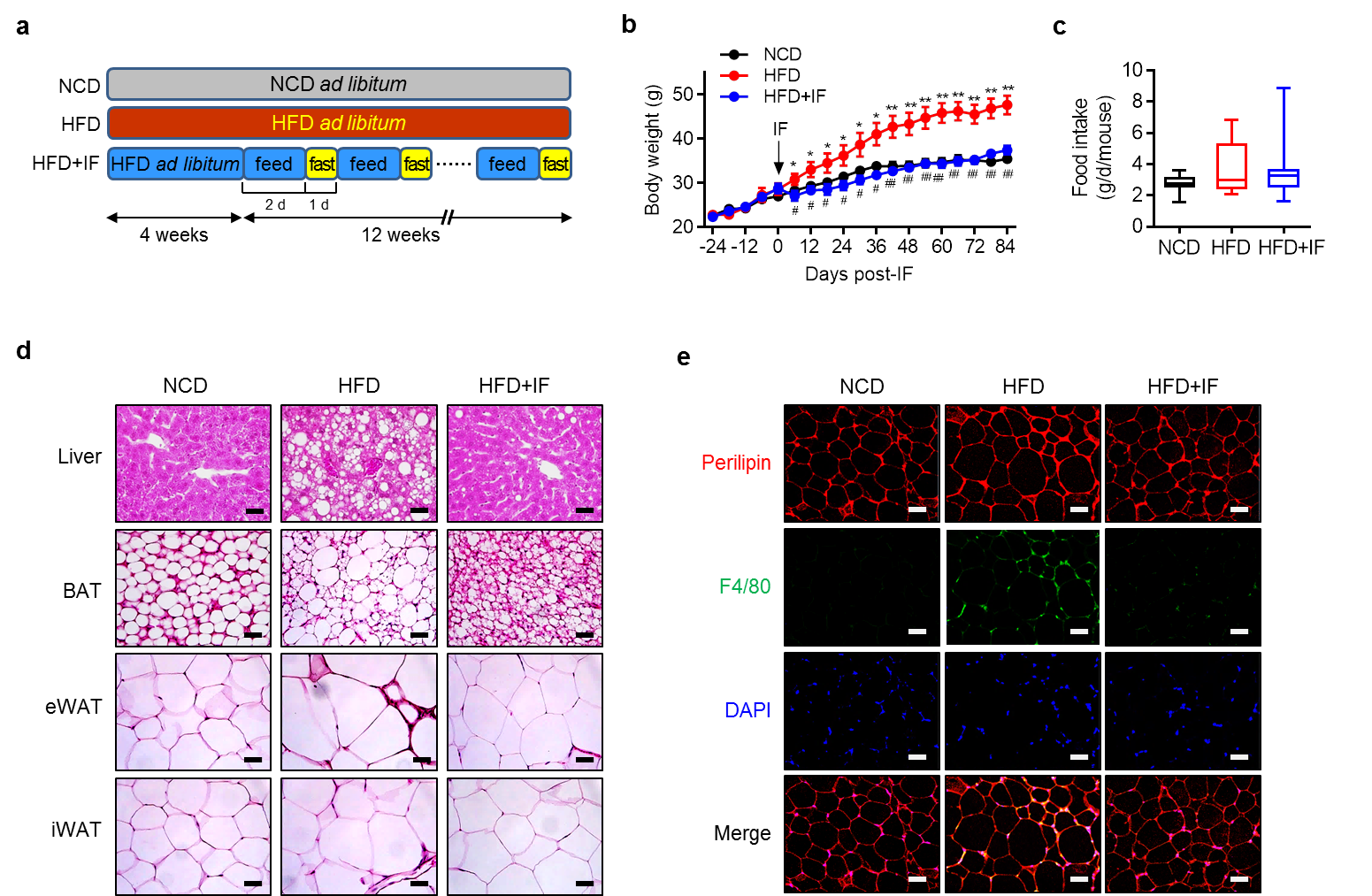


**Supplementary Figure 1.** Metabolic characteristics of intermittent HFD-fed mice. **a** Schematic overview of HFD feeding and intermittent fasting (IF) regimen. Eight-week-old mice were fed either NCD or 60% HFD *ad libitum* for 16 weeks or subjected to 12 weeks of the 2:1 IF regimen after 4 weeks of HFD *ad libitum*. **b, c** Body weight and food intake measurements during 16 weeks of diet regimen (n=5-6). **d** H&E-stained sections of liver and adipose tissues. Scale bar=25 μm. **e** eWAT was immunostained with antibodies against F4/80 and perilipin. Scar bar=50 um. Values are expressed as the mean ± SEM. *p < 0.05 and **p < 0.01 vs. NCD; #p < 0.05 and ##p < 0.01 vs. HFD+IF. NCD, normal chow diet; HFD, high fat diet; BAT, brown adipose tissue; eWAT, epididymal white adipose tissue; iWAT, inguinal white adipose tissue

**a**

**b**

**c**

**Supplementary Figure 2.** Indirect calorimetry under fed condition after16 weeks of diet regimen. **a**-**c** Oxygen consumption (VO_2_), carbon dioxide production (VCO_2_) and locomotor activity are shown (n=6).


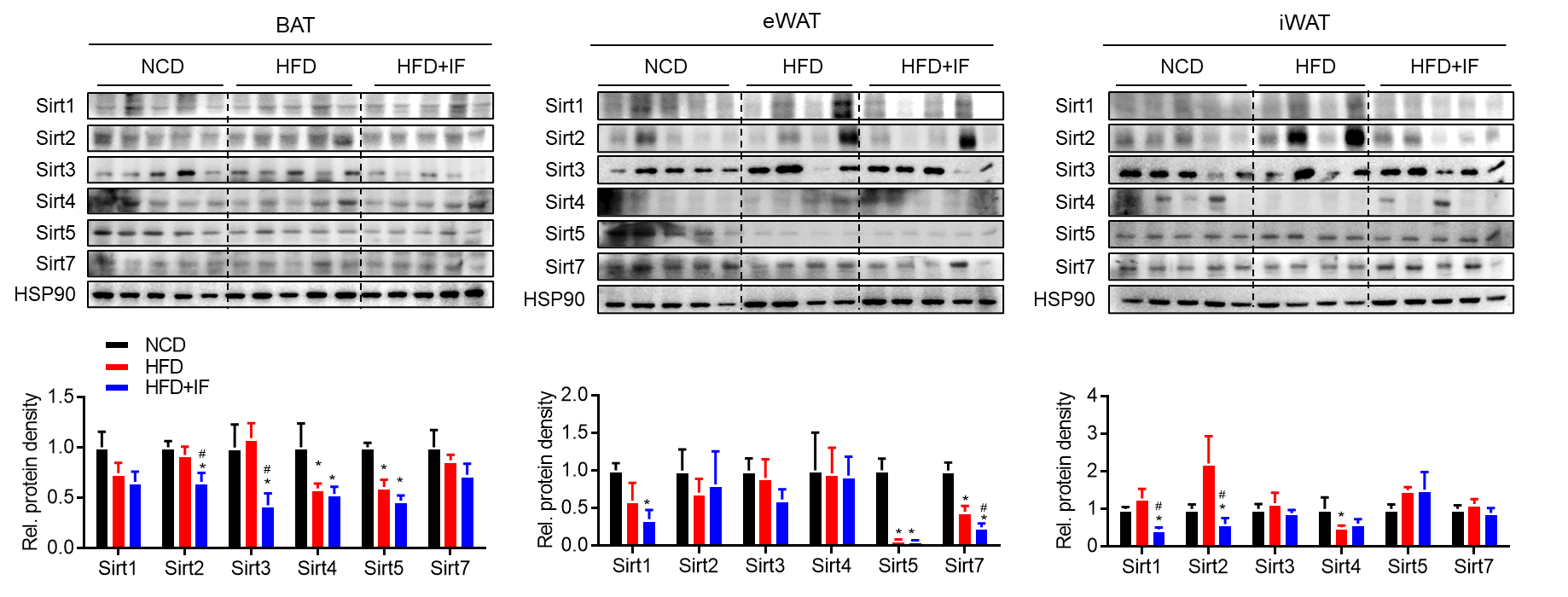


**Supplementary Figure 3.** Western blot analysis for sirtuin members in adipose tissues. Protein density was quantified (n=5) and expressed as the mean ± SEM. *p < 0.05 and **p < 0.01 vs. NCD; #p < 0.05 and ##p < 0.01 vs. HFD+IF.

**
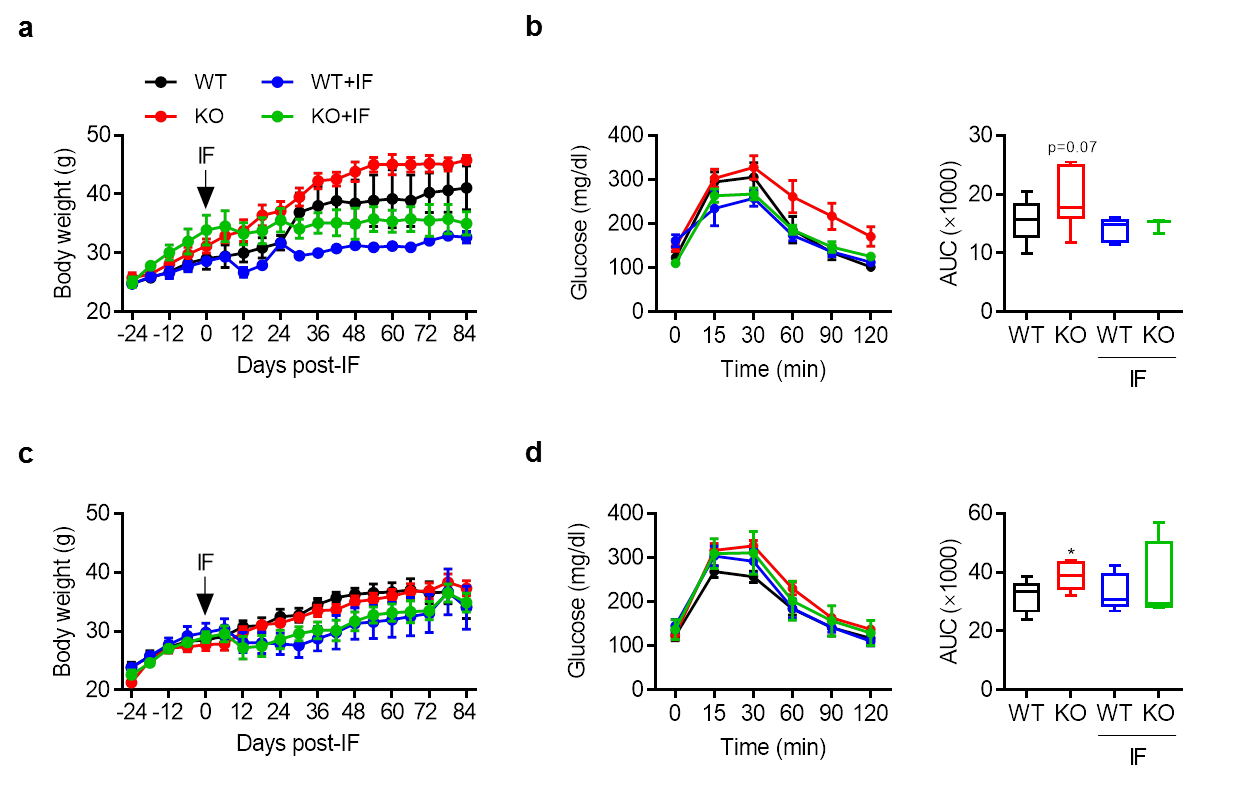
**

**Supplementary Figure 4.** Body weight changes and glucose tolerance in myeloid- and hepatocyte-specific Sirt6 KO mice. Myeloid- and hepatocyte-specific Sirt6 KO mice were generated by crossing Sirt6 floxed mice with *LysM*-cre or *Albumin*-cre mice, respectively. All experimental procedures were exactly same as described in Figure S1A. **a** Body weight measurement during 16 weeks of diet regimen in myeloid-specific Sirt6 KO mice (n=6). **b** Glucose tolerance test in myeloid-specific Sirt6 KO mice (n=6). **c** Body weight measurement during 16 weeks of diet regimen in hepatocyte-Sirt6 KO mice (n=6). **d** Glucose tolerance test in hepatocyte-Sirt6 KO mice (n=6). Values are expressed as the mean ± SEM. **p* < 0.05 vs. WT

**
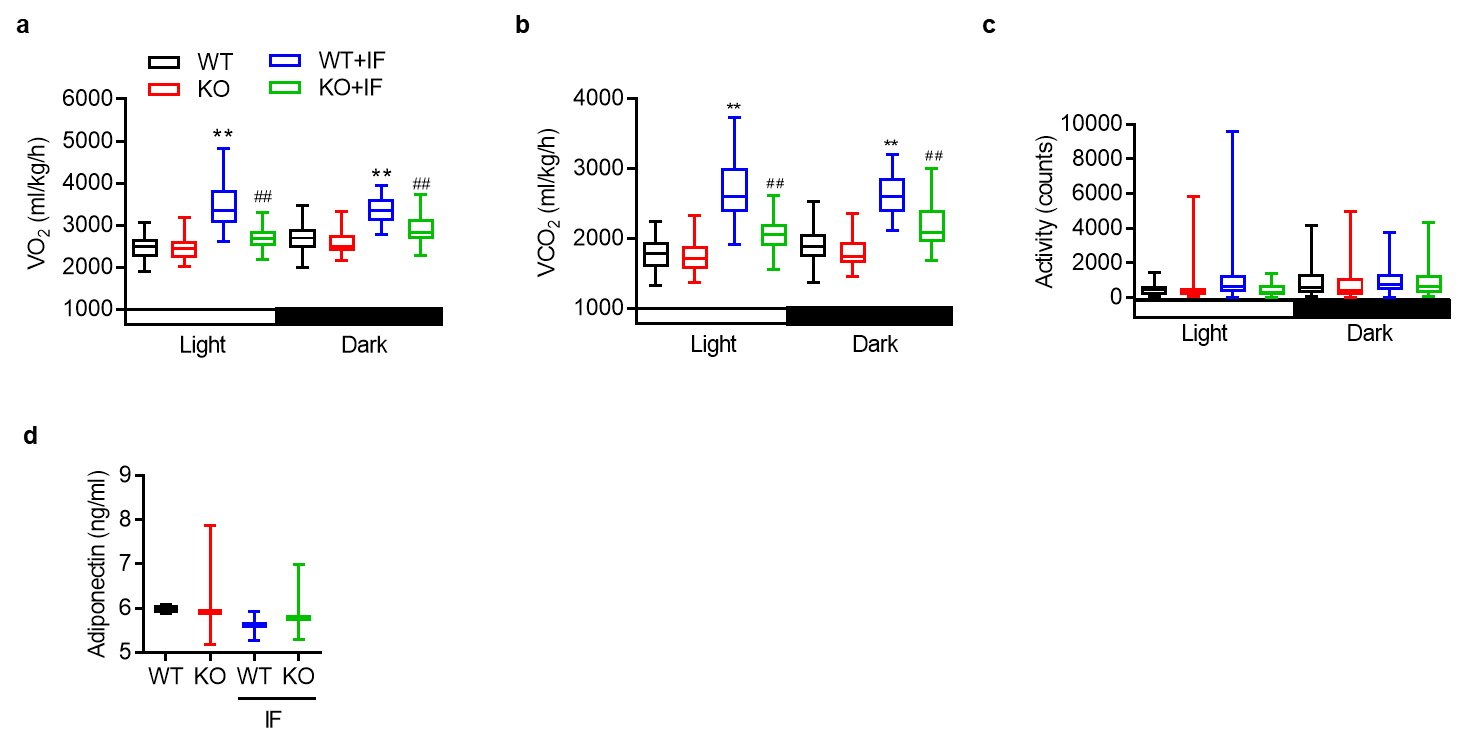
**

**Supplementary Figure 5. a-c** Indirect calorimetry under fed condition after16 weeks of diet regimen (n=4). **d** Serum level of adiponectin in WT and aS6KO mice (n=4). Values are expressed as the mean ± SEM. ^*^*p* < 0.05 and ^**^*p* < 0.01 vs. WT; ^#^*p* < 0.05 and ^##^*p* < 0.01 vs. WT+IF.

**
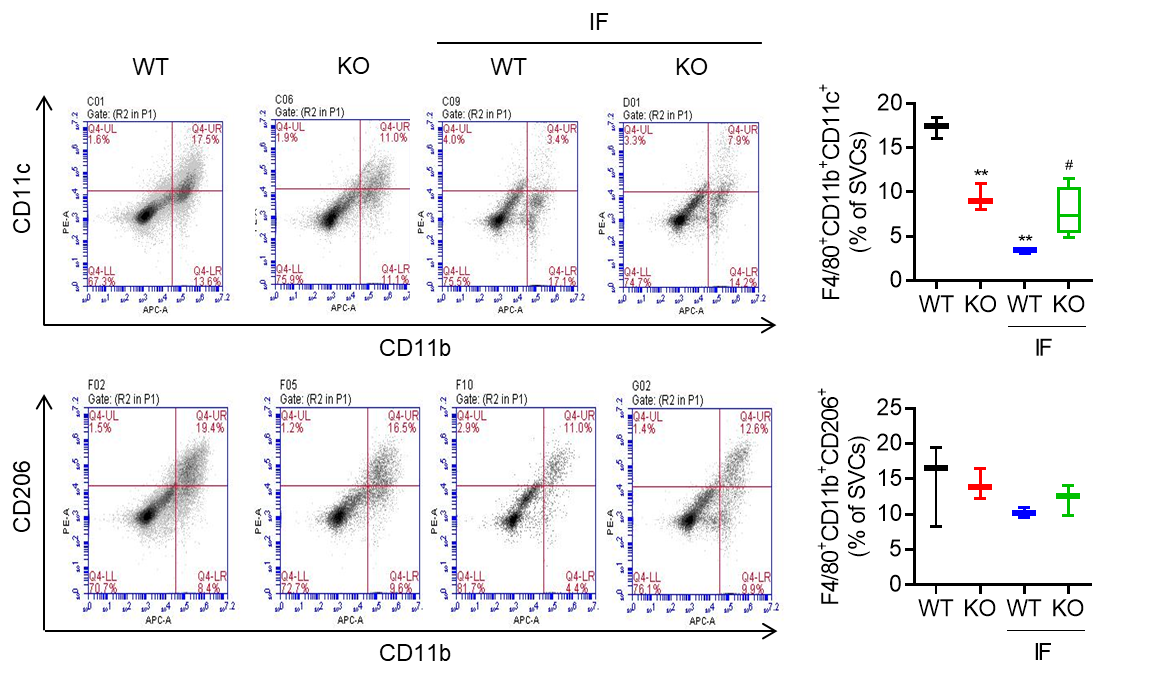
**

**Supplementary Figure 6.** Macrophage subpopulation in eWAT was analyzed by FACS analysis. Numbers of M1 (F4/80^+^CD11b^+^CD11c^+^)- or M2 (F4/80^+^CD11b^+^CD206^+^)-like macrophages were expressed as the percentage of stromal vascular cells (SVCs) (n=3). Values are expressed as the mean ± SEM. ^**^*p* < 0.01 vs. WT; ^#^*p* < 0.05 vs. WT+IF.


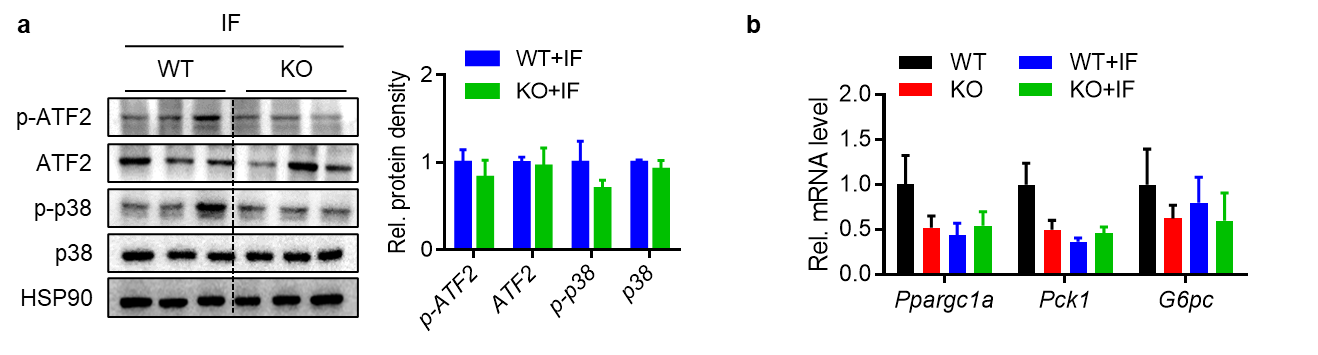


**Supplementary Figure 7. a** Western blotting analysis in BAT of WT and KO mice (n=3). **b** qPCR analysis for gluconeogenesis genes in liver (n=4). Values are expressed as the mean ± SEM.


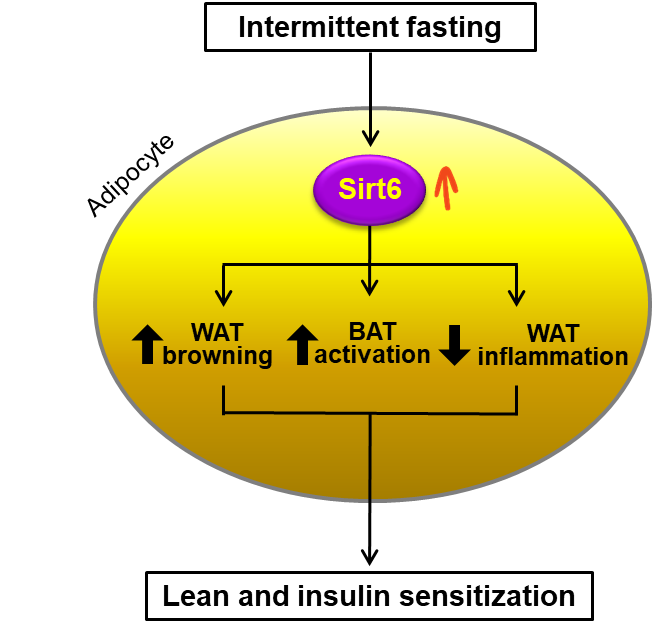


**Supplementary Figure 8. Proposed summary**
